# Sigmoidal Dependence of Electrical Conductivity of Thin PEDOT:PSS Films on Concentration of Linear Glycols as a Processing Additive

**DOI:** 10.3390/ma14081975

**Published:** 2021-04-15

**Authors:** Hyeok Jo Jeong, Hong Jang, Taemin Kim, Taeshik Earmme, Felix Sunjoo Kim

**Affiliations:** 1 School of Chemical Engineering and Materials Science, Chung-Ang University, Seoul 06974, Korea; ung2ji@daum.net (H.J.J.); qwer3197@naver.com (H.J.); skxo52@naver.com (T.K.); 2 Department of Chemical Engineering, Hongik University, Seoul 04066, Korea

**Keywords:** conducting polymer, PEDOT:PSS, electrical conductivity, processing additive, linear glycol, sigmoidal function

## Abstract

We investigate the sigmoidal concentration dependence of electrical conductivity of poly(3,4-ethylenedioxythiophene):poly(styrene sulfonate) (PEDOT:PSS) processed with linear glycol-based additives such as ethylene glycol (EG), diethylene glycol (DEG), triethylene glycol (TEG), hexaethylene glycol (HEG), and ethylene glycol monomethyl ether (EGME). We observe that a sharp transition of conductivity occurs at the additive concentration of ~0.6 wt.%. EG, DEG, and TEG are effective in conductivity enhancement, showing the saturation conductivities of 271.8, 325.4, and 326.2 S/cm, respectively. Optical transmittance and photoelectron spectroscopic features are rather invariant when the glycols are used as an additive. Two different figures of merit, calculated from both sheet resistance and optical transmittance to describe the performance of the transparent electrodes, indicate that both DEG and TEG are two most effective additives among the series in fabrication of transparent electrodes based on PEDOT:PSS films with a thickness of ~50–60 nm.

## 1. Introduction

The poly(3,4-ethylenedioxythiophene):poly(styrene sulfonate) (PEDOT:PSS) composite has been widely studied for various applications, ranging from an active layer for electronics and energy devices to functional packaging layers [1,2,3]. In optoelectronic devices, PEDOT:PSS has been used as transparent and flexible electrodes, because of its high electrical conductivity, high transmittance in the visible region, high mechanical integrity, and high ruggedness in ambient conditions [4,5,6,7,8,9]. PEDOT:PSS is also an effective buffer layers for charge injection and extraction in devices [10,11,12]. PEDOT composites can be a redox-active component for energy storage [13,14]. The high conductivity and easy control of doping states have enabled its use as a promising p-type thermoelectric material [15,16,17,18,19]. Through electrochemical doping and dedoping, it is also possible to use PEDOT composites as a conductance-controllable layer in transistor [20,21,22]. Bioelectronic applications have also been sought from the PEDOT composites [23,24]. It should be emphasized that PEDOT and the related composites are industrially compatible because they can be easily synthesized in a large scale and processed as water-based dispersions.

The electrical conductivity of PEDOT:PSS has been largely increased by varying a processing protocol in an aqueous dispersion or by applying post-deposition treatment on thin films [5,6,7,25,26,27,28,29,30,31,32,33,34]. For example, diverse classes of solvents and chemicals have been used as an additive for enhancing the electrical conductivity of PEDOT:PSS films. These additives have often been called as a secondary dopants because a minute amount is added to the stock dispersion of PEDOT:PSS, although the chemicals do not seem to significantly alter the charge-carrier density. The most noticeable chemicals include dimethyl sulfoxide (DMSO) and ethylene glycol (EG) [4,30,31,32,33,34]. DMSO and EG have been proven to enhance the conductivity and widely used for fabrication of high-conductivity electrodes. Sorbitol, which is a sugar alcohol and in a solid form at room temperature, has also been added into PEDOT:PSS for conductivity enhancement [34,35]. Surfactants have also been mentioned as an effective additive [36,37,38]. Polymers with a common building block of ethylene oxide have been mixed with PEDOT:PSS dispersions and showed a positive effect on the conductivity enhancement [39,40,41]. The governing mechanisms of conductivity enhancement have been proposed to be phase segregation between conductive parts (i.e., PEDOT) and insulating parts (i.e., PSS), bond-structural changes of PEDOT, crystallization of PEDOT, and elimination of PSS [4,26,27,28,33].

In addition to the use of additives, other methods have been implemented to increase the electrical conductivity of PEDOT:PSS composites. Dipping of PEDOT:PSS films in a solvent can remove excessive PSS and increase the conductivity [8,29,30,31,32]. Treatment with sulfuric acid (H_2_SO_4_) and organic acids has showed to be very effective in removing PSS, separating phases, and enhancing crystallinity, resulting in the electrical conductivity as high as ~4 kS/cm [6,7,42,43]. Despite the effectiveness in conductivity enhancement, it can be also challenging to apply the post-deposition treatment because the procedure may damage the underlying layers and structures.

In this work, we investigated the changes in the electrical conductivity of PEDOT:PSS films as a function of the concentrations of various linear glycols as an additive and extracted the threshold concentrations from their sigmoidal dependencies. We chose ethylene glycol (EG), diethylene glycol (DEG), triethylene glycol (TEG), hexaethylene glycol (HEG), and ethylene glycol monomethyl ether (EGME, also known as 2-methoxyethanol) as the additive, because these share common glycol structures. Although some of these chemicals have been studied before, we have focused on their concentration dependencies of systematically varied molecular structures to quantify the transition points. The electrical conductivity of the PEDOT:PSS with additives followed the sigmoidal dependence with an inflection point at the glycol concentration of ~0.6 wt.%. We then studied the optical and photoelectron spectroscopic features of the PEDOT:PSS films processed with additives and correlated the electrical properties. We also calculated two different figures of merit, which have been often used to describe the performance of the transparent electrodes, using both sheet resistance and optical transmittance of the PEDOT:PSS films. We found that both DEG and TEG could be effective in fabrication of transparent conducting electrodes based on PEDOT:PSS films with a thickness of ~50–60 nm.

## 2. Materials and Methods

An aqueous dispersion of PEDOT:PSS has the concentration of 10.2 mg/mL and the nominal weight ratio of PEDOT to PSS of 1:2.5. The PEDOT:PSS dispersion was mixed with various additives at controlled molar concentrations and stirred for 24 h at room temperature. PEDOT:PSS films were prepared on a clean glass or silicon substrate by spin-coating at 3 krpm for 60 s and annealed on a hot-plate at 140 °C for 10 min in air. The absorption spectra of the films were measured using a Jasco V-670 UV-Vis-NIR spectrophotometer. The sheet resistances (*R*_sh_) of the films were measured using 4-point probes in a colinear arrangement with a spacing of 1 mm and an Agilent 34450A digital multimeter. The resistance (*R*) in a unit of Ω, obtained by dividing the voltage difference in the inner probes by the current flowing between the outer probes, was converted to the sheet resistance (*R*_sh_) in a unit of Ω per square (Ω/sq.) by multiplying a correction factor (π/ln2) for very thin films [44]. The film thickness (*d*) was measured by using an Alpha-step 500 surface profilometer. The electrical conductivity (*σ*_dc_) was calculated with the sheet resistance and thickness of PEDOT:PSS films (*σ*_dc_ = 1/*R*_sh_*d*). At least four different points were tested to get the average values. X-ray photoelectron spectra (XPS) were obtained under ultra-high vacuum (7 × 10^−9^ mbar) by using Sigma Probe (Thermo VG Scientific, East Grinstead, UK) with a monochromatic Al-Kα X-ray at 15 kV and 100 W. Survey scan of XPS was performed at 50 eV for pass energy and 1.0 eV for step size. A high-resolution scan was performed at 20 eV for pass energy and 0.1 eV for step size. The XPS peaks were fitted with the Avantage program and calibrated with C1s (284.5 eV) as reference. Scanning electron microscopic imaging was conducted using S-3400N (Hitachi, Tokyo, Japan).

## 3. Results and Discussion

Toward the investigation of increased electrical conductivity of PEDOT:PSS film by using additives, various additives with different chemical structures have been chosen and applied. The electrical conductivity of a solidified PEDOT:PSS film is known to be increased when the stock PEDOT:PSS in a stage of aqueous dispersion is mixed with various additives such as EG, DEG, DMSO, and sorbitol [4,25,26,27,28,30,32,33,34,39]. Especially, EG is a commonly used agent for the conductivity enhancement of PEDOT:PSS since it is a liquid form at ambient conditions and is a mass-produced commercial chemical. To understand the compositional dependence of the electrical conductivity and other properties of PEDOT:PSS films on the structures of additives, we have applied EG and other linear glycol-type additives as the conductivity enhancing agents for PEDOT:PSS complexes (Table 1). These additives share common repeating units of ethylene oxide and chain ends of either hydroxyl or methoxy groups. These additives can be well mixed with water due to their hydrophilic moieties, and thus a homogeneous dispersion of PEDOT:PSS and spin-cast high-quality films can be obtained (Figure S1).

**Figure 1:**
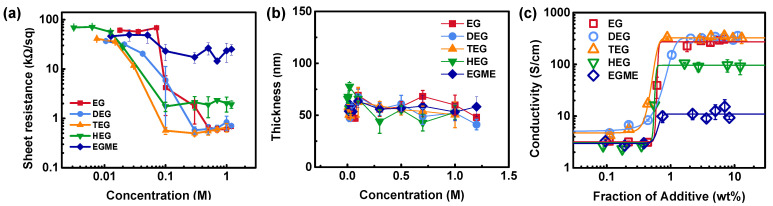
(**a**) Sheet resistance of poly(3,4-ethylenedioxythiophene):poly(styrene sulfonate) (PEDOT:PSS) films processed with linear glycol-based additives at various concentrations. (**b**) Film thickness of PEDOT:PSS films processed with the additives. (**c**) Electrical conductivity of PEDOT:PSS films as a function of the weight fraction of additives in the stock PEDOT:PSS dispersion. Solid lines in (**c**) are fitting curves of data with a sigmoidal logistic function as shown in Equation (1).

We characterized the electrical properties of the PEDOT:PSS films, which were solution-deposited after mixing with the additives at different concentrations (Figure 1). The sheet resistances (*R*_sh_) of PEDOT:PSS films were measured using a colinear 4-point-probe method. In this simple and straightforward method, the contact resistances at the film/probe interfaces can be excluded by recording the voltage difference between two inner probes under a current applied between two outer probes. The typical sheet resistance of the pristine PEDOT:PSS film was ~70–120 kΩ/sq. Figure 1a shows that the sheet resistances of the PEDOT:PSS films depend on the processing conditions. The sheet resistance of the films was clearly reduced when linear glycols were added, which also agrees to the previous studies (See Table S1) [4,22,28,32,33,34,39,40,41]. Even at a low concentration of 0.1 M of TEG, the sheet resistances dropped to 565 Ω/sq and eventually stabilized at higher concentrations. EG and DEG showed the sheet resistance transition points at slightly higher concentrations of 0.3 and 0.5 M, respectively, and the *R*_sh_ values were stabilized at 550–600 Ω/sq at higher concentrations of additives. A much longer glycol, HEG, was also effective in reducing the sheet resistance, although the stabilized sheet resistance was 2 kΩ/sq. On the other hand, EGME, which has one hydroxyl group and one methoxy group at the ends, is relatively ineffective in the resistance reduction. The sheet resistance of EGME-added PEDOT:PSS was 20 kΩ/sq.

The sheet resistance of a conductive film was also studied with the different film thickness. Figure 1b compares the thickness of the PEDOT:PSS films processed using different concentrations of the additives. All additives decreased the film thickness, compared to the average thickness of 77.7 nm of the pristine PEDOT:PSS. The thickness values are estimated around 55–60 nm at the concentration of 1.0 M. The decrease in the film thickness is similar to the previous report on the thickness of PEDOT:PSS processed with EG as an additive [32]. The decrease in thickness when processed with additives might originate from the liquid-phase additives acting as a diluting agent during the spin-coating procedure.

From the sheet resistance and thickness of the PEDOT:PSS films, we can obtain the electrical conductivity as shown in Figure 1c. The electrical conductivity represents an intrinsic property of the materials, although the sheet resistance is an important parameter of conductive films for applications in transparent electrodes. The electrical conductivity of the PEDOT:PSS films followed a sigmoidal curve as a function of the weight fraction of additives with an inflection point. The sigmoidal dependencies have been previously reported with DEG, EG, DMSO, and sorbitol, and the threshold points have been assigned to be ~0.3–0.6 wt.% [28,34]. The sigmoidal curve is observed when a cumulative effect of a certain probability distribution is important. In the case of the conductivity enhancement in PEDOT:PSS with additives, the sigmoidal behavior can be explained phenomenologically by the probability of molecular interaction between PEDOT:PSS and the additive. Then the concentrations of both polymer and additive are the determining factors for the conductivity enhancement. To quantitatively analyze the data, we have fit the data with a sigmoidal logistic function, which is a cumulative distribution function of the logistic distribution, as in Equation (1):

(1) $$\sigma_{dc}=\sigma_{0}+\frac{\sigma_{sat}-\sigma_{0}}{1+\exp\left\{-k\left(w_{add}-w_{i}\right)\right\}},$$

where *σ_dc_* is the electrical conductivity of the sample in a unit of S/cm, σ_0_ is the base conductivity of untreated PEDOT:PSS, *σ_sat_* is the saturation conductivity, *w_add_* is the weight fraction of additives in a unit of wt.% in the PEDOT:PSS dispersion, *w_i_* is a location parameter indicating the inflection point, and *k* is an inverse of a scale parameter describing the steepness of the curve. Table 2 presents the fitting results of the data with the logistic function (Equation (1)), with the coefficients of determination (*R*^2^) ranging from 0.911 for EGME to 0.997 for DEG and TEG. We note that other types of sigmoidal curves (e.g., the cumulative distribution function of the normal distribution, which can be expressed with an error function) can also describe the sigmoidal behavior. In this case, the parameters are similar to the values in Table 2 with the differences less than 7%.

The saturation conductivities are 271.8, 325.4, and 326.2 S/cm for EG, DEG, and TEG, respectively. These polar solvents can strongly interact with PSS from the PEDOT:PSS complexes, resulting in an effective phase separation in the films and/or elimination of the insulating components from the films [26,28,29,39,45]. The saturation values are much lower for HEG (95.9 S/cm) and EGME (11.0 S/cm). We believe that the low conductivity of HEG-added PEDOT:PSS films is due to the residual additives remaining in the films. It has been previously reported that the residual solvents can limit an efficient charge transport in the polymer complexes [39]. The case of EGME is interesting, considering that the structural difference between EG and EGME is one end group (hydroxyl group vs. methoxy group). Although EGME is also polar and can enhance the conductivity, the affinity with PSS is not strong enough to result in a large degree of changes. The sheet resistance of EGME-processed PEDOT:PSS films was reduced by a factor of only ~2.4 although the post-treatment was very effective in the resistance reduction by a factor of ~200 in the previous reports [5,30]. The PEDOT:PSS films have similar transition points (*w_i_*) of 0.6–1.0 wt.% of the linear glycol series as additives, which show close agreement to the values in the previous reports [28,34]. The coefficient *k*, representing how steep the curve is near the point of inflection, is the smallest for DEG. On the other hand, EG and HEG showed sharp increase in the conductivity as the additive concentration passed through the transition point. In these cases, an additive concentration of 0.8 wt.% is enough to show a saturation in the conductivity.

Such a sigmoidal dependence of the electrical conductivity on the amount of glycol additives suggests that a fraction of the components in PEDOT:PSS are influenced by the additives. The probability density function of a logistic distribution shows a symmetric curve peaked at the location parameter (i.e., *w_i_* in Equation (1)). In this case, *w_i_* may reflect the quantity of additives with the highest probability of interaction between the additive molecule and PEDOT:PSS. With a small amount of additive (*w_add_* < *w_i_*), only a small fraction of the components in PEDOT:PSS, either in an unbound form or in a complexed form, are affected and participate in molecular redistribution and reorganization. The conductivity sharply increased as the weight fraction of additives increased to near the inflection point (*w_add_* ~ *w_i_*) because many additive molecules can interact with PEDOT and PSS. When the fraction of additives increased further to the saturation points (*w_add_* > *w_i_*), there are only little molecules left to interact with. The points of inflection and saturation depend on the polymer compositions, the molecular weights, the polymerization conditions, and the degree of complexation, as well as the structure of additives.

**Figure 2:**
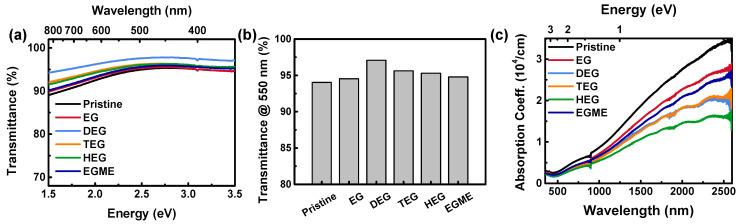
(**a**) Transmittance spectra, (**b**) representative transmittance values at 550 nm, and (**c**) absorption coefficients of the PEDOT:PSS films processed with various solvent additives at the concentrations of 1 M.

We investigated the optical properties of PEDOT:PSS films using UV-Vis-NIR spectrophotometer, since transmittance is a critical factor for applications in transparent electrodes and the spectra can also provide information on the doping states of the PEDOT composites (Figure 2) [3,8,22,46,47]. Transmittance (*T*) spectra of the PEDOT:PSS films made from dispersions with the additives (1 M), regardless of the structural variations, showed a fairly high optical transparency of *T* > 90% in the visible region with a peak value at around 450 nm. These films can be used for transparent coating layers that pass through the visible light. The transmittance *T* at 550 nm, which is often used as a benchmark condition for visually transparent coatings, was >97% obtained by addition of DEG. TEGME and EGDME follow close second and third ones, respectively.

Typical PEDOT:PSS films show a large absorption in the near-infrared (NIR) region and relatively high transmission in the visible region. Indeed, the absorption coefficient (*α*) in the NIR region reaches ~10^4^ cm^−1^, while those in the visible region were an order of magnitude lower (Figure 2c). When the additives were mixed in the PEDOT:PSS dispersion, the absorbance of films slightly decreased compared to the pristine PEDOT:PSS. However, there was no noticeable change in the shape of the absorption spectra. Although the absorption coefficients vary depending on the additives, the ratios between the absorbance at NIR to that at visible regions remain similar, suggesting that the doping states are rather invariant.

**Figure 3:**
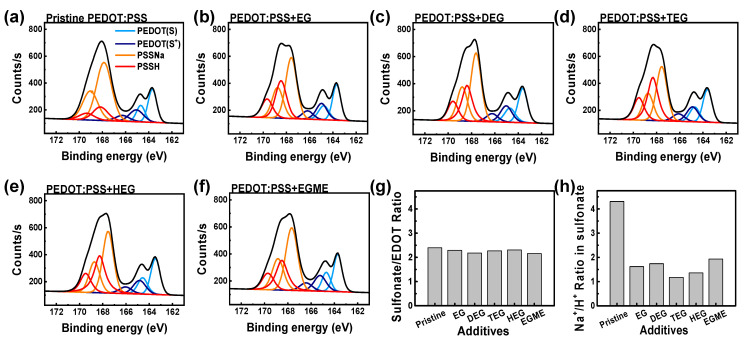
XPS S(2p) spectra of PEDOT:PSS films processed with various additives at 1 M: (**a**) Without additive and with (**b**) EG, (**c**) DEG, (**d**) TEG, (**e**) HEG, and (**f**) EGME. (**g**) Ratios of sulfonate to EDOT monomer using the areal ratios of deconvoluted peaks of the XPS signals. (**h**) Ratios of PSSNa/PSSH.

From the sulfur 2p signals of X-ray photoelectron spectroscopy (XPS), we extracted the molar ratios of sulfonate to EDOT and sodium sulfonate to sulfonic acid (Figure 3). The XPS signals for S 2p at 163–170 eV in the PEDOT:PSS films should be deconvoluted, because: (a) there are multiple peaks for the aromatic S in PEDOT with a binding energy peak at 163–166 eV and the sulfonate form of PSS at 167–170 eV, (b) each of S 2p spectra shows spin-split signals (i.e., S 2p_1/2_ and S 2p_3/2_) with the corresponding ratio of 1:2, (c) the sulfur in PEDOT can be either in a neutral form (S) or in a cationic form (S^+^), and (d) the sulfonic acid (i.e., PSSH) and sodium sulfonate (i.e., PSSNa) also have a difference of ~0.4 eV in the binding energy [26,45,48,49]. Changes in the ratios, examined by XPS, have been correlated with the conductivity increase [32,45,50]. In our samples, we found that the sulfonate-to-EDOT ratio was 2.4 for pristine PEDOT:PSS films, similar to the nominal ratio of the stock dispersion. The ratios slightly decreased to 2.29, 2.18, 2.27, 2.31, and 2.16 for EG, DEG, TEG, HEG, and EGME, respectively, as shown in Figure 3g. Because the ratios are rather close to each other, it is not clear at this moment if a change in the ratio at the surface of PEDOT:PSS films by additives is a necessary condition or not for the conductivity increase. The PSSNa/PSSH ratios dropped from 4.3 for the pristine sample to 1.6 for EG, 1.7 for DEG, 1.2 for TEG, 1.4 for HEG, and 1.9 for EGME, as shown in Figure 3h. Sodium ions can be eliminated by attractive interaction with the oxygen-rich additives.

**Figure 4:**
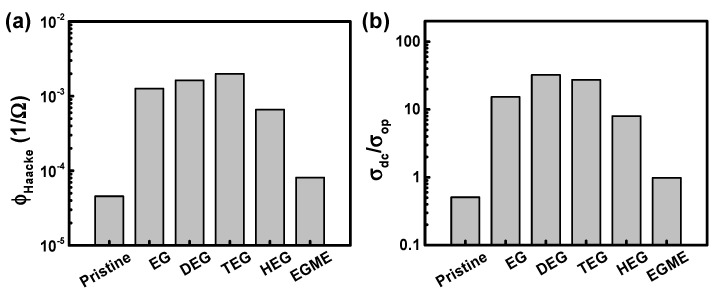
Figures of merit of the PEDOT:PSS films processed with additives. (**a**) The parameter proposed by Haacke. (**b**) The ratios of electrical conductivity to optical conductivity, at the wavelength of 550 nm.

We investigated, parameter wise, how effective these additives are for applications in transparent conducting films by comparing two representative figures of merit. Sheet resistance (*R_sh_*) and transmittance (*T*) of transparent conducting films are two important parameters and often combined to evaluate the figures of merit of transparent electrodes. One method has been proposed to use Equation (2):

(2) $$\phi_{Haacke}=\frac{T^{10}}{R_{sh}}=\sigma_{dc}d\;\exp\left(-10\alpha d\right),$$

to evaluate a figure of merit in a unit of Ω^−1^ and to quantitatively compare the performance of transparent electrodes [51]. Alternatively, the two parameters, *T* and *R_sh_*, are often correlated, at least for a thin metallic film placed in a free space, with Equation (3):

(3) $$T={\left(1+\frac{Z_{0}}{2R_{sh}}\frac{\sigma_{op}}{\sigma_{dc}}\right)}^{-1},$$

where *σ*_op_ is the optical conductivity and *Z*_0_ is the impedance of free space (*Z*_0_ = 4π/*c* = 4.19 × 10^−11^ s/cm in cgs unit, which corresponds to 337 Ω in SI unit) [46,52]. In this case, a unitless ratio of *σ_dc_*/*σ_op_* is often used as an alternative figure of merit of transparent conductive films. It has been suggested that the ratio of >10 is required for touch panels [53]. We applied these approaches to compare the effectiveness of the additives (Figure 4). We selected the wavelength of 550 nm for comparison and found that DEG and TEG are equally effective as an additive to result in the PEDOT:PSS-based transparent electrodes. The Haacke figures of merit (*Φ_Haacke_*) of the PEDOT:PSS films, calculated from Equation (2), are 1.6 × 10^−3^ Ω^−1^ for DEG and 2.0 × 10^−3^ Ω^−1^ for TEG. EG has 1.2 × 10^−3^ Ω^−1^, marking the third place among the additives of interest. For the ratios of *σ_dc_*/*σ_op_* shown in Equation (3), we get the 15.3, 32.3, and 27.3 for EG, DEG, and TEG, respectively. As expected from the electrical conductivity of the polymer films, HEG and EGME are rather ineffective as an additive for PEDOT:PSS. We note that the DEG- and TEG-added PEDOT:PSS films are more stable than the pristine PEDOT:PSS, as the films do not show significant changes in the electrical and optical properties after aging in air for several days (Figure S2).

## 4. Conclusions

In this study, we investigated the effects of linear glycols and a derivative (EG, DEG, TEG, and HEG, and EGME) as a processing additive on the electrical and optical properties of PEDOT:PSS films. We found that repeated ethylene oxides with hydroxyl groups were effective in enhancing the electrical conductivity from 3–5 S/cm in a pristine PEDOT:PSS to 270–330 S/cm with additives. We varied the concentration of the additives in the stock dispersion of PEDOT:PSS and observed sigmoidal dependence of the electrical conductivity. Through a curve fitting of the experimental conductivity–concentration data with a sigmoidal logistic function, sharp transitions were observed at the concentration of ~0.6 wt%. The PEDOT:PSS films with a thickness of 50–60 nm showed the reasonable transmittance of 94–97%. We also evaluated two different figures of merit for transparent electrodes and found that DEG and TEG among the series were effective in enhancing the performance of the PEDOT:PSS-based transparent conducting films.

## Figures and Tables

**Table 1:** Chemical structures, boiling points, and density of the additives.

Additive	Structure	Boiling Point (°C)	Density (g/cm^3^)
ethylene glycol (EG)	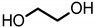	196	1.113
diethylene glycol (DEG)	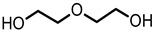	245	1.118
triethylene glycol (TEG)	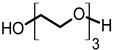	285 (4 mmHg)	1.124
hexaethylene glycol (HEG)	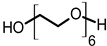	217 (4 mmHg)	1.127
ethylene glycol monomethyl ether (EGME)	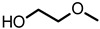	124	0.965

**Table 2:** Fitting parameters of a sigmoidal logistic function (Equation (1)).

Additive	*σ_sat_* (S/cm)	*σ*_0_ (S/cm)	*w_i_* (wt%)	*k*
EG	271.8	3.2	0.64	67.7
DEG	325.4	4.9	1.06	7.2
TEG	326.2	4.7	0.60	20.1
HEG	95.9	3.0	0.58	67.3
EGME	11.0	2.9	0.66	20.7

## Data Availability

The data presented in this study are available on request from the corresponding author.

## Supplementary Materials

The following are available online at https://www.mdpi.com/article/10.3390/ma14081975/s1. Figure S1: SEM images of the PEDOT:PSS films. Figure S2: Relative changes in the properties after aging in air. Table S1: Comparison of the film properties with previous reports.

## References

[1] Groenendaal L., Jonas F., Freitag D., Pielartzik H., Reynolds J.R. (2000). Poly(3,4-ethylenedioxythiophene) and Its Derivatives: Past, Present, and Future. Adv. Mater..

[2] Kirchmeyer S., Reuter K. (2005). Scientific importance, properties and growing applications of poly(3,4-ethylenedioxythiophene). J. Mater. Chem..

[3] Elschner A., Lövenich W. (2011). Solution-deposited PEDOT for transparent conductive applications. MRS Bull..

[4] Ouyang J., Chu C.W., Chen F.C., Xu Q., Yang Y. (2005). High-Conductivity Poly(3,4-ethylenedioxythiophene):Poly(styrene sulfonate) Film and Its Application in Polymer Optoelectronic Devices. Adv. Funct. Mater..

[5] Hsiao Y.-S., Whang W.-T., Chen C.-P., Chen Y.-C. (2008). High-conductivity poly(3,4-ethylenedioxythiophene):poly(styrene sulfonate) film for use in ITO-free polymer solar cells. J. Mater. Chem..

[6] Kim N., Kee S., Lee S.H., Lee B.H., Kahng Y.H., Jo Y.-R., Kim B.-J., Lee K. (2014). Highly Conductive PEDOT:PSS Nanofibrils Induced by Solution-Processed Crystallization. Adv. Mater..

[7] Kim S., Sanyoto B., Park W.-T., Kim S., Mandal S., Lim J.-C., Noh Y.-Y., Kim J.-H. (2016). Purification of PEDOT:PSS by Ultrafiltration for Highly Conductive Transparent Electrode of All-Printed Organic Devices. Adv. Mater..

[8] Jang H., Kim M.S., Jang W., Son H., Wang D.H., Kim F.S. (2020). Highly conductive PEDOT:PSS electrode obtained via post-treatment with alcoholic solvent for ITO-free organic solar cells. J. Ind. Eng. Chem..

[9] Jeong S.-H., Ahn S., Lee T.-W. (2019). Strategies to Improve Electrical and Electronic Properties of PEDOT:PSS for Organic and Perovskite Optoelectronic Devices. Macromol. Res..

[10] Earmme T., Jenekhe S.A. (2012). Solution-Processed, Alkali Metal-Salt-Doped, Electron-Transport Layers for High-Performance Phosphorescent Organic Light-Emitting Diodes. Adv. Funct. Mater..

[11] Cha H., Li J., Li Y., Kim S.-O., Kim Y.-H., Kwon S.-K. (2020). Effects of Bulk Heterojunction Morphology Control via Thermal Annealing on the Fill Factor of Anthracene-based Polymer Solar Cells. Macromol. Res..

[12] Wang L., Park J.S., Lee H.G., Kim G.-U., Kim D., Kim C., Lee S., Kim F.S., Kim B.J. (2020). Impact of Chlorination Patterns of Naphthalenediimide-Based Polymers on Aggregated Structure, Crystallinity, and Device Performance of All-Polymer Solar Cells and Organic Transistors. ACS Appl. Mater. Interfaces.

[13] Österholm A.M., Shen D.E., Dyer A.L., Reynolds J.R. (2013). Optimization of PEDOT Films in Ionic Liquid Supercapacitors: Demonstration As a Power Source for Polymer Electrochromic Devices. ACS Appl. Mater. Interfaces.

[14] Gulercan D., Commandeur D., Chen Q., Sarac A.S. (2019). A Ternary PEDOT-TiO2-Reduced Graphene Oxide Nanocomposite for Supercapacitor Applications. Macromol. Res..

[15] Bubnova O., Khan Z.U., Malti A., Braun S., Fahlman M., Berggren M., Crispin X. (2011). Optimization of the thermoelectric figure of merit in the conducting polymer poly(3,4-ethylenedioxythiophene). Nat. Mater..

[16] Kim G.H., Shao L., Zhang K., Pipe K.P. (2013). Engineered doping of organic semiconductors for enhanced thermoelectric efficiency. Nat. Mater..

[17] Massonnet N., Carella A., Jaudouin O., Rannou P., Laval G., Celle C., Simonato J.-P. (2014). Improvement of the Seebeck coefficient of PEDOT:PSS by chemical reduction combined with a novel method for its transfer using free-standing thin films. J. Mater. Chem. C.

[18] Park H., Lee S.H., Kim F.S., Choi H.H., Cheong I.W., Kim J.H. (2014). Enhanced thermoelectric properties of PEDOT:PSS nanofilms by a chemical dedoping process. J. Mater. Chem. A.

[19] Lee S., Kim S., Pathak A., Tripathi A., Qiao T., Lee Y., Lee H., Woo H.Y. (2020). Recent Progress in Organic Thermoelectric Materials and Devices. Macromol. Res..

[20] Bernards D.A., Malliaras G.G. (2007). Steady-State and Transient Behavior of Organic Electrochemical Transistors. Adv. Funct. Mater..

[21] Kim S.-M., Kim C.-H., Kim Y., Kim N., Lee W.-J., Lee E.-H., Kim D., Park S., Lee K., Rivnay J. (2018). Influence of PEDOT:PSS crystallinity and composition on electrochemical transistor performance and long-term stability. Nat. Commun..

[22] Kim D., Jang H., Lee S., Kim B.J., Kim F.S. (2021). Solid-State Organic Electrolyte-Gated Transistors Based on Doping-Controlled Polymer Composites with a Confined Two-Dimensional Channel in Dry Conditions. ACS Appl. Mater. Interfaces.

[23] Rivnay J., Leleux P., Ferro M., Sessolo M., Williamson A., Koutsouras D.A., Khodagholy D., Ramuz M., Strakosas X., Owens R.M. (2015). High-performance transistors for bioelectronics through tuning of channel thickness. Sci. Adv..

[24] Harman D.G., Gorkin Iii R., Stevens L., Thompson B., Wagner K., Weng B., Chung J.H.Y., in het Panhuis M., Wallace G.G. (2015). Poly(3,4-ethylenedioxythiophene):dextran sulfate (PEDOT:DS)–A highly processable conductive organic biopolymer. Acta Biomater..

[25] Huang J., Miller P.F., Wilson J.S., de Mello A.J., de Mello J.C., Bradley D.D.C. (2005). Investigation of the Effects of Doping and Post-Deposition Treatments on the Conductivity, Morphology, and Work Function of Poly(3,4-ethylenedioxythiophene)/Poly(styrene sulfonate) Films. Adv. Funct. Mater..

[26] Kim J.Y., Jung J.H., Lee D.E., Joo J. (2002). Enhancement of electrical conductivity of poly(3,4-ethylenedioxythiophene)/poly(4-styrenesulfonate) by a change of solvents. Synth. Met..

[27] Timpanaro S., Kemerink M., Touwslager F.J., De Kok M.M., Schrader S. (2004). Morphology and conductivity of PEDOT/PSS films studied by scanning–tunneling microscopy. Chem. Phys. Lett..

[28] Crispin X., Jakobsson F.L.E., Crispin A., Grim P.C.M., Andersson P., Volodin A., van Haesendonck C., Van der Auweraer M., Salaneck W.R., Berggren M. (2006). The Origin of the High Conductivity of Poly(3,4-ethylenedioxythiophene)−Poly(styrenesulfonate) (PEDOT−PSS) Plastic Electrodes. Chem. Mater..

[29] Ouyang J., Xu Q., Chu C.-W., Yang Y., Li G., Shinar J. (2004). On the mechanism of conductivity enhancement in poly(3,4-ethylenedioxythiophene):poly(styrene sulfonate) film through solvent treatment. Polymer.

[30] Martin B.D., Nikolov N., Pollack S.K., Saprigin A., Shashidhar R., Zhang F., Heiney P.A. (2004). Hydroxylated secondary dopants for surface resistance enhancement in transparent poly(3,4-ethylenedioxythiophene)–poly(styrenesulfonate) thin films. Synth. Met..

[31] Kim N., Lee B.H., Choi D., Kim G., Kim H., Kim J.-R., Lee J., Kahng Y.H., Lee K. (2012). Role of Interchain Coupling in the Metallic State of Conducting Polymers. Phys. Rev. Lett..

[32] Kim Y.H., Sachse C., Machala M.L., May C., Müller-Meskamp L., Leo K. (2011). Highly Conductive PEDOT:PSS Electrode with Optimized Solvent and Thermal Post-Treatment for ITO-Free Organic Solar Cells. Adv. Funct. Mater..

[33] Ouyang L., Musumeci C., Jafari M.J., Ederth T., Inganäs O. (2015). Imaging the Phase Separation Between PEDOT and Polyelectrolytes During Processing of Highly Conductive PEDOT:PSS Films. ACS Appl. Mater. Interfaces.

[34] Nevrela J., Micjan M., Novota M., Kovacova S., Pavuk M., Juhasz P., Kovac J., Jakabovic J., Weis M. (2015). Secondary doping in poly(3,4-ethylenedioxythiophene):Poly(4-styrenesulfonate) thin films. J. Polym. Sci. Part B: Polym. Phys..

[35] Zhang F., Johansson M., Andersson M.R., Hummelen J.C., Inganäs O. (2002). Polymer Photovoltaic Cells with Conducting Polymer Anodes. Adv. Mater..

[36] Fan B., Mei X., Ouyang J. (2008). Significant Conductivity Enhancement of Conductive Poly(3,4-ethylenedioxythiophene):Poly(styrenesulfonate) Films by Adding Anionic Surfactants into Polymer Solution. Macromolecules.

[37] Choi J.S., Yim J.-H., Kim D.-W., Jeon J.-K., Ko Y.S., Kim Y. (2009). Effects of various imidazole-based weak bases and surfactant on the conductivity and transparency of poly(3,4-ethylenedioxythiophene) films. Synth. Met..

[38] Vosgueritchian M., Lipomi D.J., Bao Z. (2012). Highly Conductive and Transparent PEDOT:PSS Films with a Fluorosurfactant for Stretchable and Flexible Transparent Electrodes. Adv. Funct. Mater..

[39] Mengistie D.A., Wang P.-C., Chu C.-W. (2013). Effect of molecular weight of additives on the conductivity of PEDOT:PSS and efficiency for ITO-free organic solar cells. J. Mater. Chem. A.

[40] Lee J.J., Lee S.H., Kim F.S., Choi H.H., Kim J.H. (2015). Simultaneous enhancement of the efficiency and stability of organic solar cells using PEDOT:PSS grafted with a PEGME buffer layer. Org. Electron..

[41] Wang T., Qi Y., Xu J., Hu X., Chen P. (2005). Effects of poly(ethylene glycol) on electrical conductivity of poly(3,4-ethylenedioxythiophene)–poly(styrenesulfonic acid) film. Appl. Surf. Sci..

[42] Xia Y., Sun K., Ouyang J. (2012). Solution-Processed Metallic Conducting Polymer Films as Transparent Electrode of Optoelectronic Devices. Adv. Mater..

[43] Ouyang J. (2013). Solution-Processed PEDOT:PSS Films with Conductivities as Indium Tin Oxide through a Treatment with Mild and Weak Organic Acids. ACS Appl. Mater. Interfaces.

[44] Uhlir A. (1955). The potentials of infinite systems of sources and numerical solutions of problems in semiconductor engineering. Bell Syst. Tech. J..

[45] Crispin X., Marciniak S., Osikowicz W., Zotti G., van der Gon A.W.D., Louwet F., Fahlman M., Groenendaal L., De Schryver F., Salaneck W.R. (2003). Conductivity, morphology, interfacial chemistry, and stability of poly(3,4-ethylene dioxythiophene)–poly(styrene sulfonate): A photoelectron spectroscopy study. J. Polym. Sci. Part B: Polym. Phys..

[46] Ellmer K. (2012). Past achievements and future challenges in the development of optically transparent electrodes. Nat. Photon..

[47] van der Pol T.P.A., Keene S.T., Saes B.W.H., Meskers S.C.J., Salleo A., van de Burgt Y., Janssen R.A.J. (2019). The Mechanism of Dedoping PEDOT:PSS by Aliphatic Polyamines. J. Phys. Chem. C.

[48] Greczynski G., Kugler T., Salaneck W.R. (1999). Characterization of the PEDOT-PSS system by means of X-ray and ultraviolet photoelectron spectroscopy. Thin Solid Films.

[49] Khan M.A., Armes S.P., Perruchot C., Ouamara H., Chehimi M.M., Greaves S.J., Watts J.F. (2000). Surface characterization of poly(3,4-ethylenedioxythiophene)-coated latexes by X-ray photoelectron spectroscopy. Langmuir.

[50] Cho H., Cho W., Kim Y., Lee J.-G., Kim J.H. (2018). Influence of residual sodium ions on the structure and properties of poly(3,4-ethylenedioxythiophene):poly(styrenesulfonate). RSC Adv..

[51] Haacke G. (1976). New figure of merit for transparent conductors. J. Appl. Phys..

[52] Dressel M., Grüner G. (2002). Electrodynamics of Solids: Optical Properties of Electrons in Matter.

[53] Hecht D.S., Hu L., Irvin G. (2011). Emerging Transparent Electrodes Based on Thin Films of Carbon Nanotubes, Graphene, and Metallic Nanostructures. Adv. Mater..

