# Supplementary material for: Sigmoidal Dependence of Electrical Conductivity of Thin PEDOT:PSS Films on Concentration of Linear Glycols as a Processing Additive
Source: Materials (Basel). 2021 Apr 15;14(8):1975. doi: 10.3390/ma14081975 (PMC8071320; doi:10.3390/ma14081975)
Supplement: Supplementary file 1 [file materials-14-01975-s001.zip › Supplementary Materials.pdf]

materials

MDPI

Supplementary Materials

Sigmoidal Dependence of Electrical Conductivity of Thin PEDOT: PSS Films on Concentration of Linear Glycols as a Processing Additive

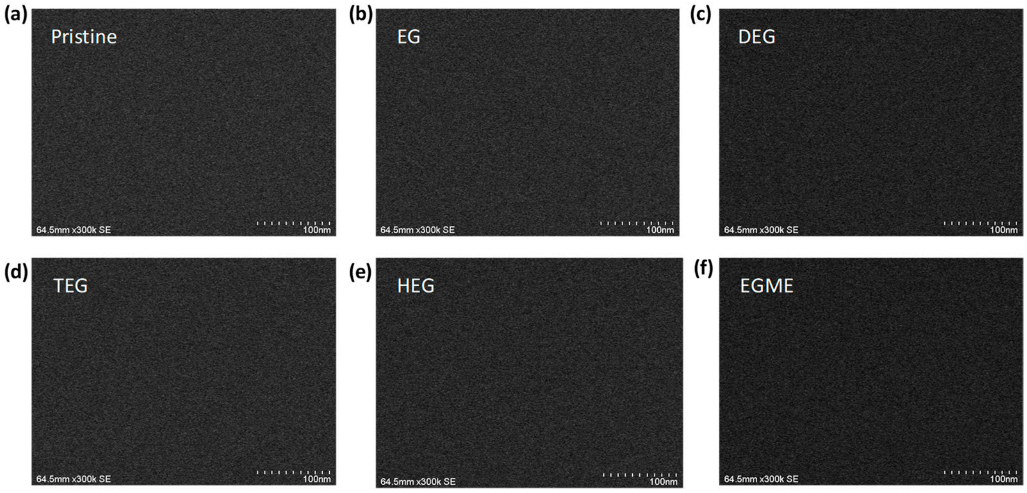

Figure S1. Scanning electron microscopy (SEM) images of the PEDOT:PSS films: (**a**) Pristine, (**b**) EG-added, (**c**) DEG-added, (**d**) TEG-added, (**e**) HEG-added, and (**f**) EGME-added samples.

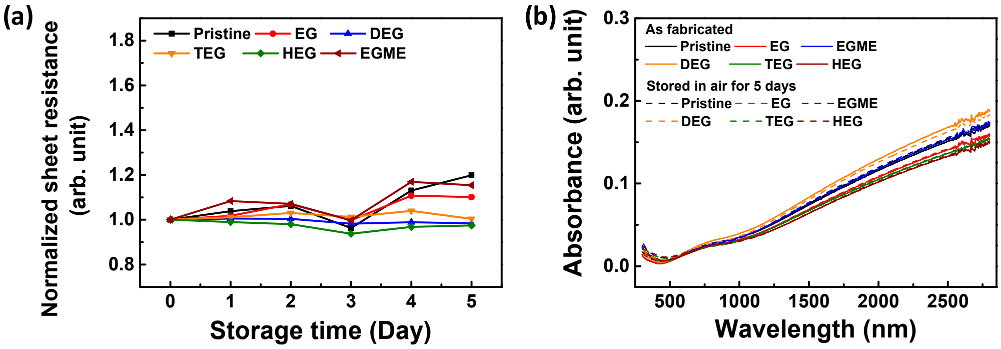

Figure S2. Relative changes in the properties after aging in air: (**a**) Sheet resistance as a function of time and (**b**) absorbance of as-fabricated samples and after 5 days.

Table S1. Comparison of the film properties with previous reports.

Additive	Composition (wt%, vol%, or mol/L)	Inflection Point (wt% or vol%)	Electrical Conductivity (S/cm)	Figure of Merit	Note	Ref.
EG	0 or 50 wt%	n/a	0.4 (at 0 wt%)<br>160 (at 50 wt%)	n/a		[1]
EG	0 or 5 wt%	n/a	5.43 (0 wt%)<br>471 (5 wt%)	n/a		[2]
DEG	0–10 wt%	0.3 wt%	0.006 (at 0 wt%)<br>~10 (at 5 wt%)	n/a		[3]
EG	0–10 vol%	~3 vol%	~1 (at 0 vol%)<br>~731 (at 6 vol%)	n/a<br>(σ_{dc}/σ_{op}=36.3 for EG dipping)	Dipping was also studied.	[4]
DEG	0 or 50 wt%	n/a	~0.1 (at 0 wt%)<br>~150 (at 50 wt%)	n/a	Polyethyleneglycol (PEG) was also studied.	[5]
PEG 400	50 wt%	n/a	~200 (at 50 wt%)	n/a		
EG	0–10 wt%	0.6 wt%	0.1 (at 0 wt%)<br>767 (at 3 wt%)	n/a		[6]
EG	0 or 6 vol%	n/a	0.3 (at 0 vol%)<br>640 (at 6 vol%)	n/a	PEGs were also studied.	[7]
EG 200	0–6 vol%	n/a	805 (at 2 vol%)	σ_{dc}/σ_{op}=39 (estimated)		
PEG 300	0–6 vol%	n/a	805 (at 2 vol%)	n/a		
PEG 400	0–6 vol%	n/a	805 (at 2 vol%)	n/a		
PEG 400	0 – 0.125 mol/L	n/a	0.1 (0 mol/L)<br>17.7 (0.04 mol/L)	n/a	Other PEGs were also studied.	[8]

References

1. Ouyang, J.; Chu, C.W.; Chen, F.C.; Xu, Q.; Yang, Y. High-Conductivity Poly(3,4-ethylenedioxythiophene): Poly(styrene sulfonate) Film and Its Application in Polymer Optoelectronic Devices. *Adv. Funct. Mater.* **2005**, *15*, 203–208, doi:10.1002/adfm.200400016.
2. Kim, D.; Jang, H.; Lee, S.; Kim, B.J.; Kim, F.S. Solid-State Organic Electrolyte-Gated Transistors Based on Doping-Controlled Polymer Composites with a Confined Two-Dimensional Channel in Dry Conditions. *ACS Appl. Mater. Interfaces* **2021**, *13*, 1065–1075, doi:10.1021/acsami.0c19006.
3. Crispin, X.; Jakobsson, F.L.E.; Crispin, A.; Grim, P.C.M.; Andersson, P.; Volodin, A.; van Haesendonck, C.; Van der Auweraer, M.; Salaneck, W.R.; Berggren, M. The Origin of the High Conductivity of Poly(3,4-ethylenedioxythiophene)−Poly(styrenesulfonate) (PEDOT−PSS) Plastic Electrodes. *Chem. Mater.* **2006**, *18*, 4354–4360, doi:10.1021/cm061032+.
4. Kim, Y.H.; Sachse, C.; Machala, M.L.; May, C.; Müller-Meskamp, L.; Leo, K. Highly Conductive PEDOT: PSS Electrode with Optimized Solvent and Thermal Post-Treatment for ITO-Free Organic Solar Cells. *Adv. Funct. Mater.* **2011**, *21*, 1076–1081, doi:10.1002/adfm.201002290.
5. Ouyang, L.; Musumeci, C.; Jafari, M.J.; Ederth, T.; Inganäs, O. Imaging the Phase Separation Between PEDOT and Polyelectrolytes During Processing of Highly Conductive PEDOT: PSS Films. *ACS Appl. Mater. Interfaces* **2015**, *7*, 19764–19773, doi:10.1021/acsami.5b05439.
6. Nevrela, J.; Micjan, M.; Novota, M.; Kovacova, S.; Pavuk, M.; Juhasz, P.; Kovac, J.; Jakabovic, J.; Weis, M. Secondary doping in poly(3,4-ethylenedioxythiophene):Poly(4-styrenesulfonate) thin films. *J. Polym. Sci. Part B: Polym. Phys.* **2015**, *53*, 1139–1146, doi:10.1002/polb.23754.
7. Mengistie, D.A.; Wang, P.-C.; Chu, C.-W. Effect of molecular weight of additives on the conductivity of PEDOT:PSS and efficiency for ITO-free organic solar cells. *J. Mater. Chem. A* **2013**, *1*, 9907–9915, doi:10.1039/C3TA11726J.
8. Wang, T.; Qi, Y.; Xu, J.; Hu, X.; Chen, P. Effects of poly(ethylene glycol) on electrical conductivity of poly(3,4-ethylenedioxythiophene)–poly(styrenesulfonic acid) film. *Appl. Surf. Sci.* **2005**, *250*, 188–194, doi:10.1016/j.apsusc.2004.12.051.
